# EEG Neurofeedback in the Treatment of Adults with Binge-Eating Disorder: a Randomized Controlled Pilot Study

**DOI:** 10.1007/s13311-021-01149-9

**Published:** 2021-12-20

**Authors:** Marie Blume, Ricarda Schmidt, Jennifer Schmidt, Alexandra Martin, Anja Hilbert

**Affiliations:** 1 Integrated Research and Treatment Center Adiposity Diseases, Department of Psychosomatic Medicine and Psychotherapy, Behavioral Medicine Research Unit, University of Leipzig Medical Center, Leipzig, Germany; 2 Muenster School of Health, FH Muenster University of Applied Sciences, Muenster, Germany; 3 Clinical Psychology and Psychotherapy, School of Human and Social Sciences, University of Wuppertal, Wuppertal, Germany

**Keywords:** Binge-eating disorder, Eating disorders, EEG neurofeedback, Beta/theta training, SCP training

## Abstract

Specific alterations in electroencephalography (EEG)-based brain activity have recently been linked to binge-eating disorder (BED), generating interest in treatment options targeting these neuronal processes. This randomized-controlled pilot study examined the effectiveness and feasibility of two EEG neurofeedback paradigms in the reduction of binge eating, eating disorder and general psychopathology, executive functioning, and EEG activity. Adults with BED and overweight (*N* = 39) were randomly assigned to either a food-specific EEG neurofeedback paradigm, aiming at reducing fronto-central beta activity and enhancing theta activity after viewing highly palatable food pictures, or a general EEG neurofeedback paradigm training the regulation of slow cortical potentials. In both conditions, the study design included a waiting period of 6 weeks, followed by 6 weeks EEG neurofeedback (10 sessions à 30 min) and a 3-month follow-up period. Both EEG neurofeedback paradigms significantly reduced objective binge-eating episodes, global eating disorder psychopathology, and food craving. Approximately one third of participants achieved abstinence from objective binge-eating episodes after treatment without any differences between treatments. These results were stable at 3-month follow-up. Among six measured executive functions, only decision making improved at posttreatment in both paradigms, and cognitive flexibility was significantly improved after food-specific neurofeedback only. Both EEG neurofeedback paradigms were equally successful in reducing relative beta and enhancing relative theta power over fronto-central regions. The results highlight EEG neurofeedback as a promising treatment option for individuals with BED. Future studies in larger samples are needed to determine efficacy and treatment mechanisms.

**Supplementary Information:**

The online version contains supplementary material available at 10.1007/s13311-021-01149-9.

## Introduction

Binge-eating disorder (BED) is characterized by recurrent episodes of binge eating, during which an objectively large amount of food is consumed, accompanied by a feeling of loss of control over eating, without regular compensatory measures to prevent weight gain (e.g., self-induced vomiting) [1]. BED is the most prevalent eating disorder with a lifetime prevalence of 2.8% in women and 1.0% in men [2]. Individuals with BED experience a significantly increased risk of lifetime obesity [3], which is defined as an excessive accumulation of body fat (body mass index; BMI ≥ 30.0 kg/m^2^) [4]. Recently, BED has been associated with changes in electroencephalography (EEG) activity, making EEG neurofeedback a possible treatment approach [5, 6].

EEG neurofeedback is a neurocognitive intervention based on the spectral analysis of the EEG. Through human–computer interaction, it enables the trainee to modify EEG parameters related to disorder-specific EEG deviations [7]. Recent research identified elevated beta activity (13 to 30 Hz) as a BED-specific EEG target [5]. Compared to individuals with obesity only, increased beta activity was found in individuals with BED over fronto-central regions in resting-state and during food cue presentation [5], with significant positive associations with eating disorder psychopathology. Elevated beta activity indicates increased awareness and attentional bias towards disorder-specific stimuli (e.g., food in BED) and can be found in a variety of mental disorders, also characterized by increased impulsiveness and decreased inhibition, for example, attention‐deficit/hyperactivity disorder (ADHD) and substance use disorders [7].

In non- and subclinical samples, randomized-controlled pilot studies evaluated the efficacy of EEG neurofeedback in the treatment of eating disorder-related behavior [8–10]. Schmidt and Martin [9, 10] applied a 10-session EEG neurofeedback with the rational of reducing high beta activity (23 to 28 Hz) over frontal brain regions to *n* = 14 and *n* = 25 restrained eaters with overeating tendencies. In these studies, it was shown that EEG neurofeedback successfully reduced food craving and overeating episodes compared to waitlist control groups (*n* = 13 and *n* = 25) and an active mental imagery group (*n* = 25). These findings were stable over 3-month follow-up. Furthermore, the authors showed that physiological learning, for example, reduction of high beta activity, occurred only in the EEG neurofeedback treatment group and was significantly related to treatment success, described as a reduction in overeating [11]. Based on observations that alpha/theta training was successful in the reduction of substance cravings in individuals with substance use disorders, Imperatori et al. [8] investigated the effects of a 10-session alpha/theta EEG neurofeedback in a healthy sample (*n* = 25) to reduce food craving compared to a waiting list control group (*n* = 25). This study demonstrated a reduction of food craving and an increase in resting-state EEG alpha activity after treatment, without effects on other frequency bands compared to waitlist controls.

To our knowledge, only one study investigated EEG neurofeedback in a clinical sample with eating disorders demonstrating its feasibility and acceptance [12]. Lackner et al. [12] conducted a randomized-controlled study, using a 10-session alpha EEG neurofeedback in women with anorexia nervosa (*n* = 10) and found posttraining improvements in eating disorder psychopathology, emotion regulation, and relative theta power during resting-state eyes closed EEG compared to a waitlist control group (*n* = 12). However, no treatment effects were observed for mood, depressive symptoms, BMI, and other frequency bands.

In the last decade, there has been much controversy regarding the role of specific cognitive abilities in the maintenance of and treatment success in BED [13–16]. Since EEG neurofeedback has proven to be efficacious for improving impulsive and disinhibited behaviors in ADHD and substance use disorders [17–19], EEG neurofeedback may have favorable effects on executive functioning in BED, which awaits further research.

In this context, the present pilot study aimed at investigating the effectiveness, feasibility, and acceptance of EEG neurofeedback in adults with BED for the first time. Specifically, this study used a food-specific paradigm, targeting high beta reduction (23 to 28 Hz) after food cue presentation. Since downtraining of any parameter is always limited by a natural zero, a parameter requiring uptraining, such as theta (5 to 7 Hz), was added to the paradigm. Uptraining of theta was deemed especially valuable in BED, based on findings that theta activity is negatively associated with cognitive control [20] and was found to be reduced in individuals with obesity during cognitively demanding tasks [5]. Slow cortical potentials (SCP) training, a general paradigm well established in treatment of ADHD [22], was used as active control intervention. We evaluated EEG neurofeedback’s potential in reducing the number of objective binge-eating episodes (OBEs) as a key diagnostic feature of BED, and improving eating disorder and general psychopathology, general executive functions, including inhibition, impulsiveness, attention, cognitive flexibility, as well as in changes in the EEG, and feasibility and acceptance of the treatment.

## Materials and Methods

### Study Design

This randomized-controlled trial used a within-between subjects design with participants being randomized to one of two active intervention groups (between-subjects), measured at four different time points (within-subjects). All participants were assessed at baseline, after the 6 weeks waiting period (pretreatment), after neurofeedback training (posttreatment), and after a 3-month follow-up period. Participants were randomized into a food-specific and a general neurofeedback condition using two stratification indices: weight status (BMI < 30 kg/m^2^ versus BMI ≥ 30 kg/m^2^) and severity of BED according to the 5th edition of the Diagnostic and Statistical Manual of Mental Disorders (DSM-5 [1]; BED full syndrome versus BED of low frequency and/or limited duration). Randomization was conducted at pretreatment by an independent researcher using an online randomization tool (http://www.randomization.com). Participants were blind for other possible group allocations, but it was not possible to keep the experimenter blind during treatment, assessment, and analysis. In agreement with the Declaration of Helsinki, the study was approved by the local Ethics Committee of the University of Leipzig (143–15-20042015) and was registered at the German Clinical Trials Registry (https://www.drks.de; Identifier: DRKS00010496). Written informed consent was obtained from all participants prior to study participation and after detailed explanation of study procedures. For posttreatment and 3-month follow up, participants were offered a compensation of 15.00 €. The Consensus on the Reporting and Experimental Design of clinical and cognitive-behavioral Neurofeedback studies (CRED-nf) was followed wherever possible and is reported in Table S1 of the Supplementary Material [21].

Based on the results of Schmidt and Martin [9, 10], an a priori power analysis suggested medium to large within group effects of neurofeedback on overeating tendencies (0.89 ≤ *d* ≤ 1.6); therefore, a sample size of *n* = 18 individuals with BED was required per group to detect medium-to-large sized reductions in OBEs (*f* = 0.30) with adequate power (1 − β = 0.80). Assuming a dropout rate of 10%, our goal was to recruit *n* = 20 individuals with BED per group.

### Participants

Recruitment took place between April 2016 and September 2018 and included web-based and local advertisements. Additionally, patients, who previously finished a behavioral weight loss program at the study site, were informed about the study. Inclusion criteria for the study were DSM-5 full syndrome BED or BED of low frequency and/or limited duration, 25 ≤ BMI < 45 kg/m^2^, age 18–60 years, and sufficient German language skills. BED diagnosis was ascertained by trained psychologists using the diagnostic items of the Eating Disorder Examination interview (EDE) [22]. Exclusion criteria were bulimia nervosa, neurological disorders or medication with influence on eating behavior, weight, or executive functions (e.g., stroke, head injury), current or planned behavioral or surgical weight loss treatment over the following 6 months, bariatric surgery in the previous 24 months, participation in other interventional studies, current psychotherapy with focus on BED, current substance use disorder, pregnancy or lactation, and uncorrected vision or hearing.

### Diagnostic Sessions

Participants were invited to four assessments (baseline, pretreatment, posttreatment, and 3-month follow-up), each containing the diagnostic items of the EDE [22], a series of questionnaires assessing eating disorder and general psychopathology, and neuropsychological computerized tests assessing executive functioning. EEG was assessed at pre- and posttreatment only.

### Primary Outcome

EDE items on binge eating were used to determine the number of OBEs over the last 14 days [22]. Considering the training duration of approximately 6 weeks, the standard 28-day period of the EDE would not have been suitable to capture change, therefore, the last 14 days were used. To represent changes in OBEs, difference scores were calculated by subtracting the number of OBEs observed at baseline from the number of OBEs observed at pretreatment and posttreatment.

### Secondary Outcomes

For secondary outcomes, the following measures were assessed at baseline, pretreatment, posttreatment, and 3-month follow-up. Detailed descriptions of the measures can be found in the Supplementary Material.

Based on the EDE [22], the number of OBEs at 3-month follow-up and abstinence from binge eating (i.e., zero binge-eating episodes) over the past 14 days were assessed. To determine global eating disorder psychopathology, the Eating Disorder Examination-Questionnaire (EDE-Q) [23] and Food Craving Questionnaire-Trait-reduced (FCQ-T-r) [24] were used.

Validated self-report measures were applied to assess self-efficacy, perceived stress, depressive symptoms, quality of life, and impulsiveness, using the General Self-Efficacy Scale (GSE) [25], Perceived Stress Questionnaire (PSQ) [26], Patient Health Questionnaire-Depression (PHQ-D) [27], Impact of Weight on Quality of Life-Lite (IWQOL-Lite) [28], and the Barratt Impulsiveness Scale short form (BIS-15) [29].

BMI and waist-to-hip ratio (WHR) were calculated using objectively measured body weight, height, and hip and waist circumference.

Regarding executive functions, decision making, cognitive flexibility, impulsivity, planning, inhibitory control, and attention were assessed using the following computerized tests: Iowa Gambling Task (IGT) [30], Wisconsin Card Sorting Test (WCST) [31], Delay Discounting Task (DDT) [32], Tower of London (TOL) [33, 34], visual Go/NoGo paradigm [34, 35], and the test battery for perception and attention functions (WAFA) [34, 36].

To determine changes in the EEG spectrum, an EEG was conducted pre- and posttreatment for 180 s eyes-open and eyes-closed resting-state as well as during 90 s food cue presentation. Out of 70 food pictures from a food pics database [37], 13 individually salient food pictures were chosen, according to a rating on likelihood of a given food being part of an OBE. For detailed information on the measures, see Supplementary Material.

The feasibility and acceptance of both EEG neurofeedback paradigms was determined through dropout rates and a satisfaction survey after each EEG neurofeedback session.

### Treatments

#### Treatment Setup

Participants received 10 individual EEG neurofeedback sessions. Each session lasted approximately 1 h, including 30-min active training. The aim was to train twice a week in the first 4 weeks and once a week during weeks 5 and 6. All participants were asked not to eat 2 to 3 h before each session to reduce between-subject variance in food deprivation. The training was conducted by the main author and five extensively trained psychologists (B. Sc.) using a standardized treatment manual.

The NEURO PRAX® EEG—full-band DC-EEG Bio- and Neurofeedback-System by Neurocare (THERA PRAX® neuroConn GmbH, Ilmenau, Germany) was used for EEG neurofeedback. The trainer, seated opposite of the trainee behind the trainer screen, did not interrupt the training unless necessary, but praised the participant after each accomplished trial to ensure motivation. Participants of both groups were not provided with a strategy. The training took place in a quiet, semi-lit room.

#### Food-Specific EEG Neurofeedback

**Fig. 1:**
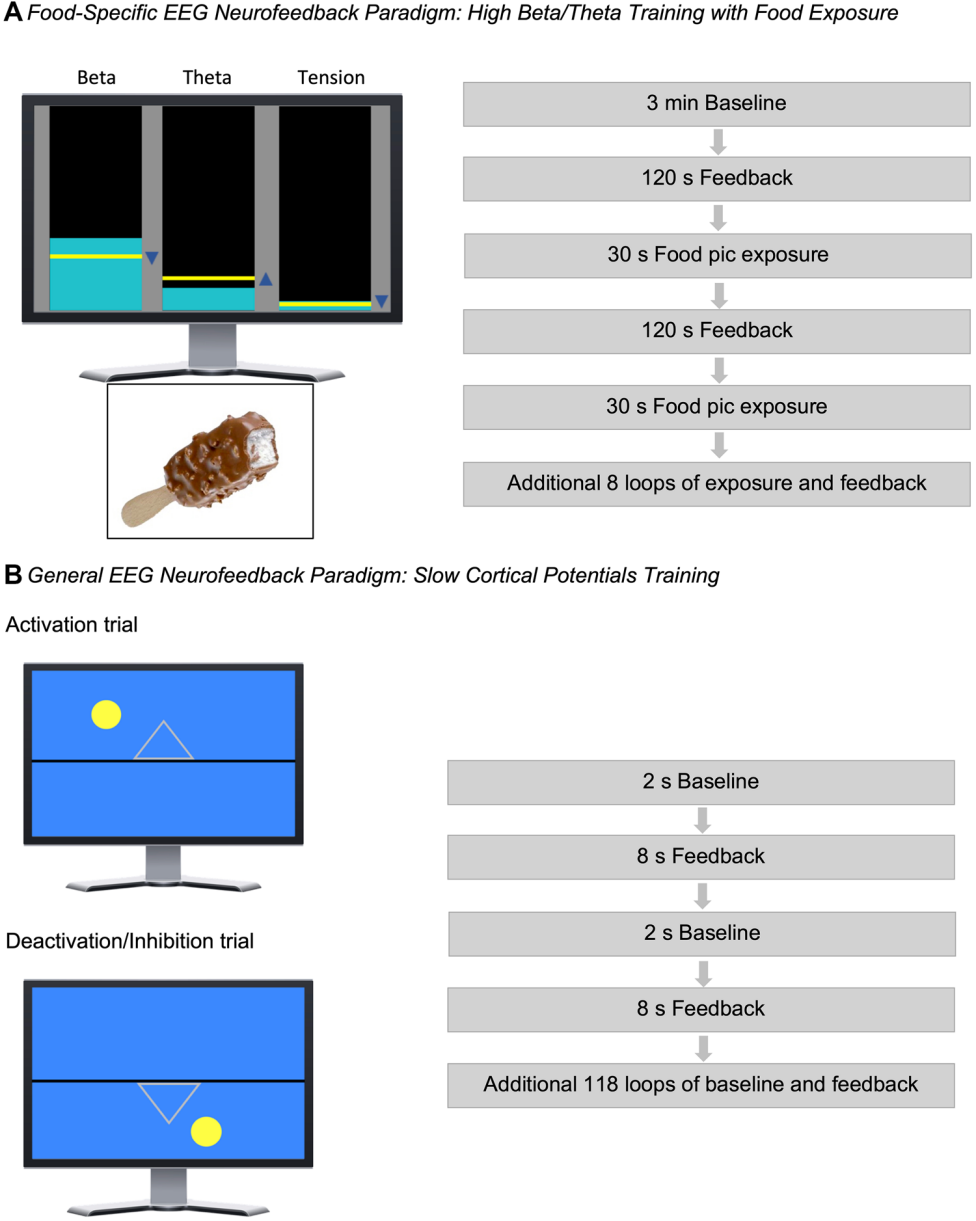
EEG neurofeedback paradigms. **A** Beta, 23–28 Hz, theta, 5–7 Hz. Tension represents muscular activity 60–80 Hz. Arrows beside bars indicate intended direction of training. Yellow lines represent the threshold of successful reduction/enhancement. Turquoise bars are constantly updated and show real time activity of each frequency band. Exemplary food picture provided by food pic database [37]. **B** Activation trial trains cortical activation associated with electrical negativation of slow cortical electrical deflections, illustrated by a yellow ball moving upward. Inhibition trial trains cortical inhibition associated with electrical positivation of slow cortical electrical deflections, illustrated by the yellow ball moving downward

Based on the findings of our systematic literature review [5] and the findings by Schmidt and Martin [9, 10], the EEG neurofeedback protocol aimed at reducing fronto-central high beta activity (23 to 28 Hz) while increasing theta activity (5 to 7 Hz). The EEG was derived from the training sites Fz, Cz, Fc1, and Fc2 in reference to the mastoids with a sampling rate of 256 samples per second (sps). A fast Fourier transformation was used to determine the activity over the fronto-central area. Eye-movements were controlled by an Electrooculogram (EOG), and low and high bandpass filters were implemented. The feedback consisted of 11 trials in total, without transfer trials. Figure 1a depicts the food-specific paradigm. Each session started with an adaption phase of 180 s, during which the individual baseline of each parameter was estimated and used as individual threshold for the session. Eleven alternating phases of self-regulation (120 s) and food presentation (30 s) followed, starting with a self-regulation phase without prior food presentation. Food pictures were selected based on the likelihood of a given food being part of an OBE. Craving (vivid imagination of food pics) and regulation phases were separated and followed subsequently during the training in order to avoid dual task interference [38]. On the black training screen, three bar diagrams were displayed, conveying the following information to the trainee: (1) a yellow fixed horizontal line, indicating the individual threshold of the frequency band, (2) an arrow beside the bar, indicating the intended direction of training, and (3) a constantly updated turquoise bar, indicating the real time activity of each frequency band.

#### General EEG Neurofeedback

As active comparison condition, a general paradigm, SCP training was used, which is well established in the treatment of ADHD [18, 39]. SCP training targets the ability to self-regulate cortical activation and inhibition [18]. In line with the standard SCP protocol, Cz was used as training site, which was referenced to the mastoids with a sampling rate of 128 sps. Since SCP training is sensitive to eye movements, an electrooculogram (EOG) real-time correction was implemented. Additionally, low and high bandpass filters were employed. Figure 1b summarizes the general paradigm. This feedback consisted of 120 trials, including 20% transfer trials without feedback animation. SCP training consisted of three alternating phases. At the beginning of each trial, a 2 s baseline was measured, evaluating the current brain activity, followed by either an 8 s activation or inhibition trial. In an activation trial, represented by a triangle pointing upwards, a cortical activation associated with electrical negativation of slow cortical electrical deflections should be achieved, illustrated by a yellow ball moving upward. Similarly, in an inhibition trial, a cortical inhibition associated with electrical positivation of slow cortical electrical deflections should be realized, illustrated by the yellow ball moving downward.

### Data Analysis

Data were prepared and analyzed using Microsoft Excel version 16.36, Brain Vision Analyzer version 2.0, IBM SPSS Statistics version 24, and R version 3.6.1. Missing data for dependent variables at posttreatment and 3-month follow-up were imputed using the missForest package in R [40]. The primary analysis was conducted on a modified intent-to-treat (ITT) sample including all participants who completed at least one EEG neurofeedback session. Additionally, all analyses were repeated on the complete case sample (CC), consisting of individuals completing all 10 EEG neurofeedback sessions and follow-up measures. In order to depict changes in outcome variables, difference scores for pretreatment, posttreatment, and 3-month follow-up in reference to the baseline were calculated, with higher scores indicating improvements for all outcome measures where positive difference scores supported the expected treatment effect (self-efficacy, decision making, cognitive flexibility, computerized impulsiveness, planning). Negative difference scores indicated improvements for measures where reductions displayed expected outcomes (OBEs, eating disorder psychopathology, food craving, stress, depression, impact of weight on quality of life, BMI, WHR, subjective impulsiveness, inhibition, alertness). Data preparation included tests of normality and sphericity. All tests were two-tailed and considered significant when *p* values were < 0.05.

A repeated measures analysis of variance (rmANOVA) was used to investigate changes in the number of OBEs from baseline to pre- and posttreatment (using difference scores pre- and posttreatment) separately for both groups. For secondary outcomes, we evaluated group-specific treatment effects for long-term reductions in the number of OBEs and eating disorder psychopathology, general psychopathology, inhibition, impulsivity, attention, and cognitive flexibility using a time × group rmANOVA with time as within-subjects factor (difference scores pretreatment, posttreatment, follow-up), group as between-subjects factor (food-specific, general paradigm), and time × group interaction. In case of a significant time effect, a Bonferroni-corrected post hoc test was conducted. Between-group differences in abstinence from OBEs as categorical variable at posttreatment and 3-month follow-up were tested with *χ*^2^ tests. Exploratively, *χ*^2^ tests were used to examine within-group changes in abstinence from OBEs between posttreatment and 3-month follow-up. Changes in relative EEG power during resting-state eyes-open, eyes-closed, and during food picture presentation were separately analyzed using a time × group rmANOVA (within subjects: pretreatment, posttreatment; between subjects: food-specific, general paradigm). EEG analysis was only applied on data sets offering a minimum of 30 artifact-free segments of filtered EEG; therefore, the overall sample size varied across conditions. In case of violation of normality and sphericity, non-parametric tests were used and reported if results differed from the parametric test results. In case of a violation of sphericity, Huynh–Feldt, if ε > 0.75, or Greenhouse–Geisser, if ε < 0.75, correction were used [41]. As effect size for rmANOVAs, partial eta squared (η_p_^2^) was used, whereby values ≥ 0.01 refer to small, ≥ 0.06 to medium, and ≥ 0.14 to large effects [42]. For mean differences in post hoc tests Cohen’s *d* was calculated, with *d* ≥|0.2| being considered a small effect, *d* ≥|0.5| a moderate, and *d* ≥|0.8| a large effect [42]. For binary variables, the phi coefficient (φ) was used as effect size, with φ ≥|0.1| being considered a small effect, φ ≥|0.3| a moderate, and φ ≥|0.5| a large effect [42].

## Results

**Fig. 2:**
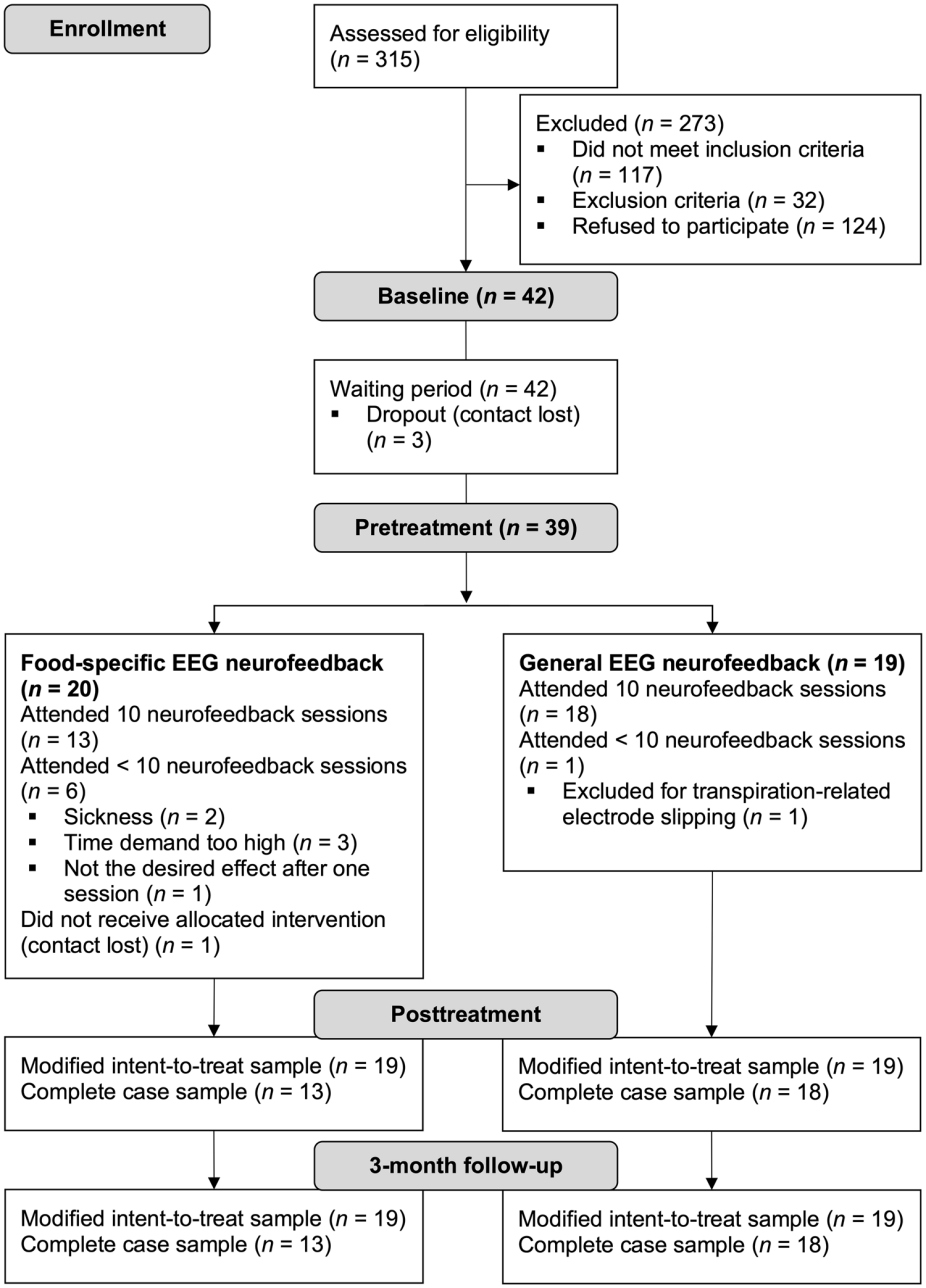
Consolidated Standards of Reporting Trials (CONSORT) flowchart of participants. Randomization was conducted by an independent researcher at pretreatment

Detailed participant flow is depicted in the Consolidated Standards of Reporting Trials (CONSORT) diagram, see Fig. 2. Overall, *n* = 36 participants had a full-threshold DSM-5 diagnosis of BED, *n* = 2 individuals had a DSM-5 diagnosis of BED of low frequency and/or limited duration, equally distributed among both neurofeedback groups. Further information on participant flow, demographics and pretreatment values can be found in the Supplementary Material.

### Primary Outcome

**Fig. 3:**
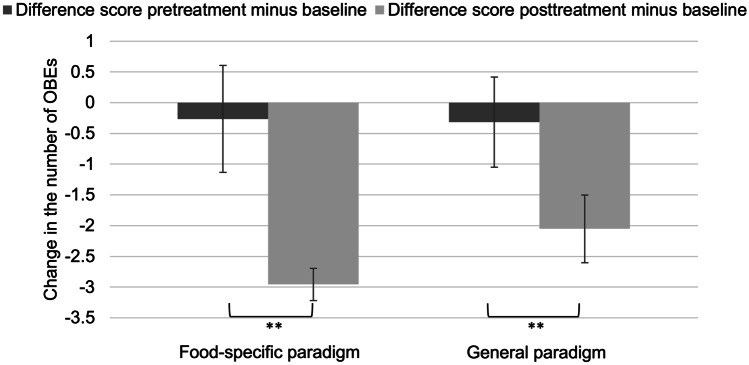
Treatment effects of EEG neurofeedback on number of binge-eating episodes. OBEs: Objective binge-eating episodes over the past 14 days. Mean and standard error are depicted. **p < .01

The rmANOVA indicated a significant, large-sized reduction of OBEs at posttreatment versus pretreatment in the food-specific paradigm, *F*(1, 18) = 8.71, *p* < 0.01, η^2^ = 0.33. In the general paradigm, results indicated a significant reduction in the number of OBEs at posttreatment compared to pretreatment with a large time effect, *F*(1, 18) = 10.78, *p* < 0.01, *η*^2^ = 0.38. Changes in the primary outcome are displayed in Fig. 3.

CC analysis revealed similar results, indicating significant reductions of OBEs at posttreatment compared to pretreatment in both paradigms (see Supplementary Tables S3 and S4).

### Secondary Outcomes

For the number of OBEs, a significant time effect emerged, but group or time × group effects were nonsignificant, see Tables 1 and 2. Post hoc analysis indicated that the number of OBEs stayed significantly reduced from posttreatment to 3-month follow-up across groups, corresponding to a medium effect, −2.18, 95% CI [−4.16, −0.19], *p* = 0.03, *d* = −0.57. In the food-specific paradigm, 31.6% of participants were abstinent from OBEs at posttreatment and 26.3% were abstinent at 3-month follow-up, which represents a nonsignificant change from posttreatment to 3-month follow-up, χ^2^(1, *N* = 18) = 2.22, *p* = 0.14, φ = 0.35. In the general paradigm, 36.8% abstained from OBEs at posttreatment and 52.6% did so at 3-month follow-up, indicating a significant change from posttreatment to 3-month follow-up, *χ*^2^(1, *N* = 19) = 4.87, *p* = 0.03, φ = 0.51. Nevertheless, posttreatment and 3-month follow-up abstinence rates did not differ significantly between groups, *χ*^2^(1, *N* = 38) = 0.12, *p* = 0.73, φ = − 0.06, and *χ*^2^(1, *N* = 37) = 2.37, *p* = 0.12 φ = −0.25, respectively.

**Table 1:** Means and standard deviations for outcome variables of the modified intent-to-treat sample

Variable	Food-specific neurofeedback (*n* = 19)				General neurofeedback (*n* = 19)			
	Baseline	Δ Pretreatment	Δ Posttreatment	Δ 3 months	Baseline	Δ Pretreatment	Δ Posttreatment	Δ 3 months
Clinical measures								
Objective binge-eating episodes	4.32 (3.64)	−0.26 (2.56)	−2.96 (3.90)	−2.33 (4.66)	4.32 (2.77)	−0.32 (3.73)	−2.05 (3.06)	−2.61 (4.58)
Eating disorder psychopathology	3.06 (1.03)	−0.13 (0.59)	−0.35 (0.78)	−0.45 (0.97)	2.83 (1.08)	− 0.08 (0.58)	−0.14 (0.75)	−0.55 (0.83)
Food craving	58.89 (11.15)	−1.58 (10.88)	−11.11 (14.22)	−10.90 (20.44)	62.21 (12.01)	−4.00 (8.29)	−12.93 (10.41)	−19.05 (13.87)
Self-efficacy^a^	28.32 (4.73)	−0.05 (3.85)	−0.12 (3.18)	0.52 (3.03)	24.74 (4.94)	0.21 (3.08)	1.24 (2.43)	2.02 (3.18)
Perceived stress	42.19 (18.22)	3.86 (16.85)	4.73 (14.85)	2.11 (15.09)	52.19 (19.37)	−1.23 (13.65)	−1.81 (18.51)	−2.42 (21.83)
Depressive symptoms	7.42 (3.27)	0.68 (2.50)	0.04 (3.42)	0.16 (3.33)	9.11 (4.15)	0.58 (4.91)	−0.65 (4.93)	0.14 (6.05)
Impact of weight on quality of life	108.26 (14.76)	3.79 (8.89)	6.25 (11.27)	5.52 (12.89)	108.35 (12.77)	0.06 (7.53)	2.40 (13.72)	7.20 (13.56)
Subjective impulsivity	30.02 (6.73)	0.40 (3.22)	1.24 (4.44)	1.41 (4.90)	35.45 (5.70)	1.44 (4.56)	1.56 (5.93)	1.18 (5.11)
Body mass index	36.65 (5.71)	0.01 (0.65)	−0.04 (2.72)	−1.08 (3.18)	35.60 (4.40)	−0.99 (2.97)	−0.02 (1.41)	−0.56 (1.80)
Waist-to-hip ratio	0.86 (0.08)	0.02 (0.04)	0.02 (0.06)	0.02 (0.06)	0.90 (0.09)	0.00 (0.04)	0.00 (0.06)	−0.01 (0.05)
Executive functions								
Decision making^a^	−4.21 (36.05)	−4.95 (27.84)	3.35 (37.48)	18.92 (34.31)	4.95 (35.58)	17.26 (41.14)	25.44 (31.56)	27.38 (42.30)
Cognitive flexibility^a^	0.02 (4.09)	−1.92 (4.75)	0.95 (4.33)	2.39 (5.17)	2.17 (8.74)	−2.82 (8.70)	−1.18 (8.39)	−1.81 (8.89)
Impulsivity^a^	0.45 (0.28)	0.05 (0.14)	0.14 (0.16)	0.14 (0.21)	0.57 (0.26)	0.08 (0.15)	0.07 (0.20)	0.10 (0.18)
Planning^a^	15.63 (3.69)	1.37 (3.40)	1.01 (2.85)	1.03 (3.66)	17.63 (2.99)	−0.21 (3.33)	0.82 (3.25)	−0.16 (3.11)
Inhibitory control	10.05 (7.79)	−1.69 (6.40)	−2.57 (7.15)	−1.28 (8.44)	10.32 (5.00)	−2.65 (3.49)	−2.69 (4.91)	−2.45 (4.15)
Alertness	1.82 (1.07)	0.03 (1.32)	−0.37 (1.09)	−0.19 (1.24)	2.17 (1.31)	−0.50 (1.64)	−0.06 (2.07)	−0.39 (1.62)

*N* = 38. Δ, Difference score of pre-, posttreatment, 3-month follow-up minus baseline. Objective binge-eating episodes: Eating Disorder Examination; eating disorder psychopathology: Eating Disorder Examination-Questionnaire (0–6* less favorable scores are asterisked); food craving: Food Cravings Questionnaire-trait-reduced (15–90*); self-efficacy: General Self-Efficacy Scale (10*–49); perceived stress: Perceived Stress Questionnaire (0–100*); depressive symptoms: Patient Health Questionnaire-Depression (0–27*); impact of weight on quality of life: Impact of Weight on Quality of Life-Lite (31–155*); subjective impulsivity: Barratt Impulsiveness Scale (15–60*); decision making: Iowa Gambling Task; cognitive flexibility: Wisconsin Card Sorting Test; impulsivity: Delay Discounting Task, planning: Tower of London; inhibitory control: visual Go/NoGo paradigm

^a^Positive difference values represent effects in expected direction

**Table 2:** Repeated measures univariate analysis of variance in the modified intent-to-treat sample

Variable	Time			Group			Time x Group		
	*df*	*F*	η_p_^2^	*df*	*F*	η_p_^2^	*df*	*F*	η_p_^2^
Clinical measures									
Objective binge-eating episodes	2, 46	8.97**	.20	1, 36	0.03	.00	2, 46	0.55	.02
Eating disorder psychopathology	2, 72	5.84**	.14	1, 36	0.07	.00	2, 72	0.95	.03
Food craving	2, 56	22.23**	.38	1, 36	1.17	.03	2, 56	1.68	.05
Self-efficacy	2, 57	2.12	.06	1, 36	1.81	.05	2, 57	0.67	.02
Perceived stress	2, 57	0.29	.01	1, 36	1.26	.03	2, 57	0.10	.00
Depressive symptoms	2, 72	1.83	.05	1, 36	0.04	.00	2, 72	0.27	.01
Impact of weight on quality of life	2, 63	2.53	.07	1, 36	1.81	.05	2, 63	1.28	.03
Subjective impulsivity	2, 72	0.33	.01	1, 36	0.08	.00	2, 72	0.52	.01
Body mass index	2, 63	1.30	.04	1, 36	0.10	.00	1, 63	1.25	.03
Waist-to-hip ratio	2, 52	0.87	.02	1, 36	2.79	.07	2, 52	0.07	.00
Executive functions									
Decision making	2, 72	5.02**	.12	1, 36	3.12	.08	2, 72	1.09	.03
Cognitive flexibility	2, 64	11.68**	.25	1, 36	1.24	.03	2, 64	3.97*	.10
Impulsivity	2, 72	2.14	.06	1, 36	0.34	.01	2, 72	1.40	.04
Planning	2, 72	0.59	.02	1, 36	1.14	.03	2, 72	1.23	.03
Inhibitory control	2, 72	0.60	.02	1, 36	0.18	.02	2, 72	0.32	.01
Alertness	2, 72	0.08	.00	1, 36	0.10	.00	2, 72	2.29	.06

*N* = 38. Objective binge-eating episodes: Eating Disorder Examination; eating disorder psychopathology: Eating Disorder Examination-Questionnaire; food craving: Food Cravings Questionnaire-trait-reduced; self-efficacy: General Self-Efficacy Scale; perceived stress: Perceived Stress Questionnaire; depressive symptoms: Patient Health Questionnaire-Depression; impact of weight on quality of life: Impact of Weight on Quality of Life-Lite; subjective impulsivity: Barratt Impulsiveness Scale; decision making: Iowa Gambling Task; cognitive flexibility: Wisconsin Card Sorting Test; impulsivity: Delay Discounting Task, planning: Tower of London; inhibitory control: visual Go/NoGo paradigm

^*^p < .05; **p < .01

Global eating disorder psychopathology and food craving improved over time reflected by a significant, large-sized time effect in the absence of any group and time × group effects. For global eating disorder psychopathology, post hoc Bonferroni tests revealed a small, non-significant effect of time at posttreatment, *p* = 0.77, *d* = −0.22, and a medium, significant effect at 3-month follow-up compared to pretreatment, −0.39, 95% CI [−0.72, −0.06], *p* = 0.02, *d* = −0.66. Likewise, for food craving, post hoc analysis demonstrated significant, large time effects at posttreatment, −9.23, 95% CI [−13.37, −5.10], *p* < 0.01, *d* = −1.08, and at 3-month follow-up compared to pretreatment, −12.19, 95% CI [−18.12, −6.25], *p* < 0.01, *d* = −1.35. Other secondary outcomes including self-efficacy, perceived stress, depressive symptoms, impact of weight on quality of life, subjectively measured impulsiveness, as well as anthropometrical measures were not significantly impacted by time or group or their interaction, see Table 2.

Regarding executive functions, a significant, large-sized time effect was found for improved decision making in complex and uncertain situations (IGT), without any group or time x group effects. Post hoc analysis indicated a significant, large-sized improvement at 3-month follow-up compared to pretreatment, 16.99, 95% CI [2.79, 31.19], *p* = 0.01, *d* = 0.50, but not at posttreatment in both groups, 8.24, 95% CI [−4.58, 21.05], *p* = 0.35, *d* = 0.26. For cognitive flexibility (WCST), a significant, medium-sized time × group interaction was found, *p* = 0.02, *η*^2^ = 0.10, qualifying a large main effect of time, *p* < 0.01, *η*^2^ = 0.25, while the group effect was nonsignificant, *p* = 0.27, *η*^2^ = 0.03. The food-specific neurofeedback group learned better from their mistakes in successive trials of the WCST at posttreatment (*M* = 0.95, *SD* = 4.33) and at 3-month follow-up (*M* = 2.39, *SD* = 5.17), whereas the general neurofeedback group performed worse at posttreatment (*M* = -1.18, *SD* = 8.39) and at 3-month follow-up (*M* = -1.81, *SD* = 8.89), when compared with pretreatment. As shown in Table 2, impulsivity, planning, inhibition, and attention were not influenced by time, group, or time × group effects.

The CC analysis revealed the same pattern of results as the modified ITT analysis for secondary outcomes (see Supplementary Tables S3 and S4), with only one exception: the time effect for decision making in complex and uncertain situations (IGT) did not reach significance, when the CC sample was analyzed, *p* = 0.09, *η*^2^ = 0.08.

#### EEG Brain Activity

In the resting-state eyes-closed condition (*n* = 20), there was a significant, large-sized reduction of beta activity and a significant, medium-sized increase of theta activity over time, while no significant time effect for alpha activity was observed, see Table 3. No significant main effect for group or interaction of time × group was observed. In the resting-state eyes-open condition (*n* = 21), a significant, large time effect was found in the increase of alpha activity and in the decrease of beta activity, whereas no time effect was found for theta, while group and the time × group interaction were nonsignificant. During food presentation (*n* = 29), there was a significant and medium-sized time effect in the increase of alpha activity, a significant and large-sized time effect in the decrease of beta activity, and no significant time effect in theta activity. Again, no significant main effect for group or for time × group interaction was observed.

**Table 3:** Relative EEG band power in % in eyes-open, eyes-closed, and during food presentation at pre- and posttreatment

Variable	Food-specific neurofeedback *M (SD)*		General neurofeedback *M (SD)*		Time			Group			Time × group		
	Pretreatment	Posttreatment	Pretreatment	Posttreatment	*df*	*F*	η_p_^2^	*df*	*F*	η_p_^2^	*df*	*F*	η_p_^2^
Eyes-closed	*n* = 12	*n* = 12	*n* = 18	*n* = 18									
Alpha	32.18 (16.43)	32.92 (17.92)	30.29 (9.53)	31.24 (10.48)	1, 28	0.12	.00	1, 28	0.03	.00	1, 28	0.01	.00
Beta	25.21 (11.37)	4.78 (3.40)	25.54 (7.30)	3.99 (0.98)	1, 28	849.21**	.97	1, 28	0.05	.00	1, 28	0.28	.01
Theta	15.17 (4.66)	16.17 (5.30)	16.19 (4.02)	18.18 (4.73)	1, 28	4.46*	.14	1, 28	1.14	.04	1, 28	0.40	.01
Eyes-open	*n* = 13	*n* = 13	*n* = 18	*n* = 18									
Alpha	22.82 (10.70)	26.69 (12.19)	19.29 (6.95)	20.40 (6.82)	1, 29	15.43**	.35	1, 29	1.17	.04	1, 29	2.76	.09
Eyes-open	*n* = 13	*n* = 13	*n* = 18	*n* = 18									
Beta	28.92 (11.10)	5.87 (3.95)	27.84 (7.52)	5.51 (1.15)	1, 29	646.62**	.96	1, 29	0.09	.00	1, 29	0.46	.02
Theta	18.14 (3.99)	17.15 (3.61)	18.77 (4.56)	19.18 (3.73)	1, 29	0.29	.01	1, 29	1.07	.31	1, 29	2.92	.09
Food presentation	*n* = 11	*n* = 11	*n* = 18	*n* = 18									
Alpha	17.48 (7.68)	18.80 (7.35)	17.25 (5.69)	17.66 (4.79)	1, 27	4.24*	.14	1, 27	0.00	.00	1, 27	0.74	.03
Beta	31.68 (10.59)	6.97 (4.49)	27.48 (6.24)	5.75 (1.21)	1, 27	707.86**	.96	1, 27	0.96	.03	1, 27	0.06	.00
Theta	17.99 (4.45)	18.24 (4.18)	18.02 (4.47)	18.96 (3.60)	1, 27	2.23	.08	1, 27	0.10	.00	1, 27	0.60	.02

*N* differs because only EEG datasets with > 30 sequences were analyzed. Alpha = 8–12 Hz, beta = 13–30 Hz, theta = 4–7 Hz

^*^p < .05; **p < .01

#### Feasibility and Acceptance

In the food-specific paradigm, 68.4% (*n* = 13) of participants, who started the treatment, completed all 10 sessions, while in the general paradigm, 94.7% (*n* = 18) completed all 10 sessions. Reasons for dropping out were sickness, changing time demands at work, or relocation to another city. Both EEG neurofeedback paradigms were well accepted, without significant group differences, *F*(1, 31) = 2.16, *p* = 0.15, *η*^2^ = 0.07. Further information on duration and acceptance are presented in the Supplementary Material.

## Discussion

To our knowledge, this is the first study to investigate effectiveness, feasibility, and acceptance of EEG neurofeedback in the treatment of adult BED. It was shown that a 10-session food-specific and general EEG neurofeedback paradigm were effective in reducing the number of OBEs, the key clinical feature of BED, without one paradigm being superior to the other. Treatment effects were maintained at 3-month follow-up. Participants of both groups showed significant improvements in food craving posttreatment and significant improvements in global eating disorder psychopathology, food craving, and decision making at 3-month follow-up. Cognitive flexibility was improved in the food-specific paradigm only, at posttreatment and 3-month follow-up. Notably, both EEG neurofeedback paradigms altered EEG activity at posttreatment, indicating specific treatment-induced physiological changes. Furthermore, EEG neurofeedback treatment was highly accepted and feasible in adults with BED. Adults with BED did not report discomfort or adverse events caused by the treatment.

A major achievement of this first pilot study on EEG neurofeedback in adult BED was the significant, large-sized posttreatment reduction of OBEs and its maintenance over the 3-month follow-up period independent of the training paradigm. Abstinence rates up to 52.6% at 3-month follow-up in the general paradigm are consistent with meta-analytic gains found for psychotherapy, the most well-established treatment approach for BED [43]. Since the general paradigm was not adapted to BED and did not include food cues, its effectiveness in reducing OBEs and inducing abstinence may reflect the efficacy of mechanism-based treatment modules in general in the treatment of BED. The improvements observed for binge-eating episodes, as well as the altered EEG activity, are in line with two EEG neurofeedback studies by Schmidt and Martin [9, 10], investigating an EEG neurofeedback paradigm aimed at the reduction of high beta activity in a sample of restrained eaters with overeating tendencies. Significant medium-sized reductions observed after EEG neurofeedback in general eating disorder psychopathology are similar to those in psychotherapy and self-help treatment [43]. The reduction of food cravings after EEG neurofeedback seems to be consistent across different samples (healthy, restrained eaters with overeating tendencies, and now BED) and different EEG neurofeedback paradigms (targeting high beta activity, alpha/theta, or slow cortical potentials) [8–10], indicating that EEG neurofeedback may facilitate improvements in food-specific self-regulatory capacities.

Other secondary outcomes such as self-efficacy, stress, depressive symptoms, and quality of life were not significantly changed by either EEG neurofeedback paradigm. These results are in line with Lackner et al. [12], who found no influence of EEG neurofeedback on depressive symptoms in anorexia nervosa, and furthermore with Schmidt and Martin [9, 10], who did not detect changes caused by EEG neurofeedback on perceived stress, dietary, or somatic stress. Due to low baseline depressive symptomatology with scores between 5 and 10 on the PHQ-D in the present study, a significant improvement in depressive symptomatology was hardly achievable. Regarding nonsignificant effects of anthropometric measures, the treatment period of 6 weeks might have been too short to obtain measurable changes in BMI and WHR. Nevertheless, our results are consistent with other neurofeedback studies, including a pilot study by Chirita-Emandi and Puiu [44] showing that in a sample of adolescents with obesity, BMI standard deviation scores were not significantly reduced by 20 sessions of infra-low-frequency neurofeedback training.

Notably, two of six assessed executive functions showed significant changes after treatment, although they were not directly trained. Significant improvements in decision making were found in both EEG neurofeedback paradigms. Svaldi et al. [45] found that individuals with BED engaged significantly more often in disadvantageous decision making than weight-controlled individuals without BED. The authors argued that advantageous decision making is important in the periods preceding OBEs in BED. Only participants in the food-specific paradigm, but not the general paradigm, showed an improvement in the WCST [31]. This may indicate increased cognitive flexibility and might enable participants to be more flexible in dealing with food cravings in everyday life. Further studies are needed to investigate potential mechanisms of action and explain paradigm-specific treatment effects, for example, on associations between changes in EEG activity and cognitive control/working memory after high beta/theta EEG neurofeedback [46, 47]. However, since WCST and IGT were performed repeatedly and parallel tests were unavailable, training effects may have occurred. The fact that other executive functions, such as planning, attention, inhibition, and impulsivity were not changed by EEG neurofeedback, may be related to low numbers of training sessions. In ADHD, 30 to 40 EEG neurofeedback sessions were found to be necessary to improve symptoms of hyperactivity [18].

At posttreatment, both EEG neurofeedback paradigms were equally successful in reducing relative beta activity in the eyes-open resting-state, eyes-closed resting-state, and food condition, and enhance relative theta activity in the eyes-closed resting-state over fronto-central regions. The achieved changes in EEG activity seem to reflect an improvement in disorder-related EEG activity rather than a paradigm-specific effect, since beta reduction and theta enhancement were not specifically trained in the general paradigm. A study investigating effects of SCP neurofeedback in children with ADHD by Liechti et al. [48], suggests that SCP neurofeedback leads to a normalization of EEG patterns, which may explain a training-induced decrease of beta activity in the general paradigm. Heightened beta activity over frontal areas has been discussed as characteristic biomarker for addictions [49, 50], and has been associated with rewarding stimuli, such as drug cues in substance use disorders [51]. While theta is commonly associated with working memory maintenance and cognitive control [20], enhancements in this frequency band may indicate positive effects on cognition in individuals with BED through various EEG neurofeedback paradigms. Lackner et al. [12] also found increased resting-state eyes-closed relative theta activity after EEG neurofeedback aimed at enhancing alpha power in individuals with anorexia nervosa. In the present study, it was found that as soon as eyes were opened, the effect on theta disappeared, and we saw significant enhancements in relative alpha power (resting-state eyes-open and food cue presentation), which is in line with a 16-session EEG neurofeedback paradigm aiming at reducing relative beta1 power (12–22 Hz) over frontal and parietal areas in healthy individuals [52]. Beyond artificial band limits, general treatment effects of EEG neurofeedback on alpha enhancements were discussed in this context [52]. Alternatively, higher alpha activity in prefrontal regions might be related to higher working memory capacity and cognitive control [46].

### Strengths and Limitations

Among the strengths of this study were (1) the randomized-controlled design, (2), within-group comparisons with the untreated waiting period, (3) between-group comparisons with an active control group, (4) interview-based diagnosis of BED, (5) 3-month follow-up, (6) investigation of changes in EEG posttreatment, (7) consideration of computerized tests examining executive functioning, (8) modified ITT analysis to address attrition, and (9) the CRED-nf was followed and reported.

As limiting factors, the small sample size has to be mentioned. As this study was a pilot study, group sizes were too small to investigate between-group differences or small effects with adequate power. Follow-up studies in larger samples would allow for more in-depth analyses with mixed models to account for individual trajectories as well. Furthermore, only four electrodes located at the prefrontal region were used to assess pre- and post-EEG changes. Nevertheless, changes in alpha activity are most frequently seen in parietal and parieto-occipital regions, future studies are recommended to use a full head EEG in order to evaluate effects on alpha activity more comprehensively. In order to determine effects and mechanism of change more precisely, future studies are recommended to evaluate treatment effects on specific subranges of beta activity (i.e., beta 1 versus beta 2). Since research indicated decreased inhibitory control during food-specific tasks in individuals with BED [53, 54], we recommend future studies to use not only general inhibitory assessments, but add food-related assessments using food-related executive function tasks, in order to investigate changes regarding food-specific inhibitory control. Although a positive effect on OBEs was observed after 10 sessions of EEG neurofeedback, it would be desirable to offer a higher number of sessions, which was related to better treatment outcomes in ADHD [18]. EEG recordings were only carried out pre- and posttreatment, therefore no conclusion can be drawn about long-term EEG changes.

### Implications and Conclusion

This study uniquely demonstrated the effectiveness, acceptance and feasibility of EEG neurofeedback in the treatment of BED. A food-specific as well as a general EEG neurofeedback paradigm yielded significant reductions of binge eating and associated psychopathology. As these results were stable at 3-month follow-up, EEG neurofeedback may be considered as promising treatment option for individuals with BED. Both EEG neurofeedback paradigms were well accepted by the participants. Given the absence of significant group differences regarding primary and secondary outcomes, it is of note that participants undergoing the general paradigm showed significant improvements in abstinence from OBEs from posttreatment to 3-month follow-up, while participants receiving the food-specific paradigm did not. Considering that BED is a complex mental disorder involving both cognitive and emotional processes [55], we suggest to investigate possible benefits of using EEG neurofeedback as add-on for psychotherapy rather than as stand-alone therapy, to further increase remission rates in the treatment of BED, for example, in cognitive-behavioral therapy [43]. Future studies in larger samples are needed to determine treatment mechanisms to optimize, further adapt, and refine EEG neurofeedback in the treatment of BED, for example, using different types of cue presentation (sequential versus superimposed format).

## Supplementary Information

Below is the link to the electronic supplementary material.

Supplementary file1 (DOCX 70 KB)

Supplementary file2 (PDF 485 KB)

Supplementary file3 (PDF 509 KB)

Supplementary file4 (PDF 508 KB)

Supplementary file5 (PDF 214 KB)

Supplementary file6 (PDF 490 KB)
