# Supplementary material for: EEG Neurofeedback in the Treatment of Adults with Binge-Eating Disorder: a Randomized Controlled Pilot Study
Source: Neurotherapeutics. 2021 Dec 20;19(1):352–65. doi: 10.1007/s13311-021-01149-9 (PMC9130382; doi:10.1007/s13311-021-01149-9)
Supplement: Supplementary file 1 — Supplementary file1 (DOCX 70 KB) [file 13311_2021_1149_MOESM1_ESM.docx]

# Supplementary Material for:

Blume, M., Schmidt, R., Schmidt, J., Martin, A., & Hilbert, A. EEG Neurofeedback in the Treatment of Adults with Binge-Eating Disorder: A Randomized Controlled Pilot Stud

## Supplementary Text

1. Supplementary Methods
2. Supplementary Results

## Supplementary Tables

**Table S1.** *Consensus on the Reporting and Experimental Design of clinical and cognitive- behavioral Neurofeedback studies (CRED-nf) Checklist*

**Table S2.** *Sociodemographic Characteristics of the Modified Intent-to-Treat*

**Table S3.** *Sample Means and Standard Deviations for Outcome Variables of the Complete Case Sample*

**Table S4.** *Repeated Measures Univariate Analysis of Variance for the Complete Case Sample*

## References

# Supplementary Text

## 1. Supplementary Methods

### Psychological Measures

**Eating Disorder Psychopathology.** The 28-item Eating Disorder Examination-Questionnaire (EDE-Q) was used to determine participants’ global eating disorder psychopathology over the past 28 days [1]. The questionnaire uses a 7-point Likert scale. The global mean score was calculated ranging between 0 and 6, with higher values representing greater eating disorder psychopathology, encompassing aspects of restrained eating, eating concern, weight concern, and shape concern. In this study’s sample, internal consistency was α = .90.

**Abstinence.** Abstinence from binge eating was described as zero objective binge-eating episodes (OBEs) over the past two weeks assessed with the Eating Disorder Examination interview (EDE) [2].

**Food Craving.** The 15-item Food Cravings Questionnaire-Trait-reduced (FCQ-T-r) was used to measure food craving on a 6-point Likert scale [3]. The total sum score ranging between 15 and 90 was used to indicate food cravings, with higher scores representing greater food cravings. Internal consistency in this study’s sample was α = .91.

**Self-Efficacy.** The 10-item General Self-Efficacy Scale *(*GSES) was used to measure global self-confidence in coping with challenging situations on the basis of one's own competence on a 4-point Likert scale [4]. Higher total sum scores (10 to 40) indicated greater general self-efficacy. In this study’s sample, internal consistency was α = .89.

**Perceived Stress.** The 20-item short version of the Perceived Stress Questionnaire (PSQ) was used to measure subjectively perceived stress [5]. The PSQ distinguishes four subscales: worries, tension, joy, and demands, and each item is scored on a 4-point Likert scale. The total sum score ranging from 0 to 100 was calculated, with higher scores indicating more subjectively perceived stress. In this study’s sample, internal consistency was α = .92.

**Depressive Symptoms.** The nine depression items of the short version of the Patient Health Questionnaire (PHQ-D) were used to rate depressive symptoms over the last 2 weeks on a 4-point Likert scale [6]. A higher global sum score ranging from 0 to 27 indicates more severe depression. Internal consistency in this study’s sample was α = .79.

**Impact of Weight on Quality of Life.** The 31-item Impact of Weight on Quality of Life-Lite (IWQOL-Lite) questionnaire was used to measure obesity-related quality of life during the last week [7]. The questionnaire assesses five domains: physical function, self-esteem, sexual life, public distress, and work, using a 5-point Likert scale. Higher overall sum scores (31 to 155) indicated poorer quality of life in terms of a greater impact of weight on quality of life. Internal consistency in this study’s sample was α = .83.

**Impulsiveness.** To assess impulsive tendencies by self-report, the 15-item short form of the Barratt Impulsiveness Scale (BIS-15) was used [8]. The BIS-15 assesses three dimensions of impulsivity: non-planning, motor, and attentional impulsivity. The overall sum score (15 to 60) was used as a general measure for impulsive tendencies, with higher scores indicating subjectively higher impulsive tendencies. In this study’s sample, internal consistency was α = .86.

**Anthropometric Measures.** Using calibrated instruments, the body mass index (BMI, kg/m^2^) and the waist-to-hip ratio (WHR, cm) were derived from objectively measured body weight, height, and hip and waist circumference.

### Comorbid mental disorders. The presence of mental disorders was assessed at baseline by a self-report questionnaire. Participants were asked to list their comorbid mental disorder diagnoses using an open-ended question.

### Neuropsychological Assessments

**Decision Making.** A computerized Iowa Gambling Task (IGT) was used to measure decision making in complex and uncertain situations [9]. The aim was to win the highest possible amount of virtual money. Therefore, a card was drawn 100 times from four possible decks (A, B, C, D). The decks contained different amounts of profits and losses. To win the maximum amount of virtual money, it was necessary to find out which deck contains long-term wins (C and D) and losses (A and B). The total net value, i.e., the difference between the total number of loss decisions and the total number of win decisions, was used as outcome measure. Higher net values indicate more favorable decision making.

**Cognitive Flexibility.** Cognitive flexibility was determined using a computerized version Wisconsin Card Sorting Test (WCST) [10]. In the WCST, a maximum of 128 cards had to be sorted according to three possible principles, for example, shape, color, or number. The rules were unknown and could only be assumed by using the provided feedback (correct versus wrong response). Once a rule was correctly determined, the rule would change suddenly and without warning after 10 consecutive and correctly sorted cards. Therefore, the participant had to flexibly adapt the sorting strategy. As outcome measure, the learning to learn score was used, representing the average growth of conceptual efficiency over consecutive trials (average difference in percent errors between successive categories). The learning to learn score can be positive or negative, with positive values representing increased efficiency across consecutive categories, presumably because of learning [10].

**Impulsivity.** A computerized Delay Discounting Task (DDT) was used to determine impulsive decision making [11]. Participants were presented with a choice between an immediate reward (variable amount between 0 and 10 Euro without delay) and a fixed delayed reward (0, 2, 30, 180 and 365 days). For each of the five temporal delays, an indifference point was calculated where the subjective value of the immediate reward and the delayed reward were the same. Performance in DDT was determined using the area under the curve (AUC) for the five indifference points. The AUC can range from 0 to 1, with larger AUC values being associated with lower discounting of delayed rewards, i.e., lower impulsivity [12].

**Planning.** The planning capability was measured with the computerized version of the Tower of London (TOL) task provided by the Vienna Test System [13, 14]. Participants were presented with a number of problems, resolving around the placement of three differently colored balls on three rods with different capacities (one, two or three balls). The three balls had to be transferred from an initial state to a target state. There were several rules to be followed, for example, every ball had to be placed on a rod at every time. The difficulty and the number of optimal moves to solve a problem increased constantly, starting from two move tasks to six move tasks. The planning ability was determined by an overall planning score. Higher values indicated higher planning abilities [13].

**Inhibitory Control.** Inhibition was assessed with a computerized visual Go/NoGo paradigm provided by the Vienna Test System [14, 15]. In this task, 250 stimuli were randomly presented, 202 stimuli required a reaction, while 48 stimuli required an inhibition. The number of commission errors (false positive responses to a No-Go trial) was used as an outcome measure, with more commission errors indicating decreased inhibitory control [16].

**Attention.** Variability in early attentional processes was measured using a computerized test-battery for perception and attention functions (WAFA), provided by the Vienna Test System [14, 17]. In a first task, an irregularly occurring visual stimulus had to be detected as quickly as possible (intrinsic alertness). In a second task, each stimulus was preceded by an acoustic signal, allowing the participant to prepare a possible reaction, ideally resulting in faster response times (phasic alertness). Variability in performance in intrinsic and phasic vigilance was reported as a measure of dispersion, with higher values indicating greater variability in intrinsic or phasic alertness [17].

### Feasibility and Acceptance

The feasibility of both electroencephalography (EEG) neurofeedback paradigms was determined by conducting a general satisfaction survey after each EEG neurofeedback training session. Participants were asked how much they liked the training on a 5-point Likert scale. The average mean score (1 to 5) was used as outcome measure, with higher scores indicating greater satisfaction.

### EEG Stimuli

**Binge Food.** To determine individually salient food stimuli, 70 food pictures were presented on a computer screen and the likelihood of the food to be part of an OBE was rated on a visual analogue scale ranging from 0 = *not likely at all* to 400 = *very likely*. The food pictures were provided by the Blechert et al.’s Food Pics Database [18]. Out of 568 possible pictures from this database, 70 images were selected on the basis of recognizability, palatability [18], variability, expert ratings provided by our research group, and frequently mentioned binge foods in EDEs of former patients with binge-eating disorder (BED). The 13 images with the highest probability of being part of a binge-eating episode were individually selected. Three of these 13 images were randomly selected for presentation during pre- and post-EEG recording for all participants. The remaining ten images were selected for presentation in the food-specific EEG neurofeedback paradigm. This rating took place at pretreatment, a few days before the start of the EEG neurofeedback training.

### EEG recordings

At the beginning of the first and last EEG neurofeedback session, an EEG was recorded in both EEG neurofeedback paradigms with three conditions: (1) 180 s resting-state eyes-open, (2) 180 s resting-state eyes-closed, and (3) 90 s exposure with 3 binge-food pictures. Using the NEURO PRAX® EEG by neurocare (THERA PRAX® neuroConn GmbH, Ilmenau, Germany) to obtain spectral EEG, four Ag/AgCl electrodes were placed at the neurofeedback training sites (Cz, Fz, Fc1, Fc2) at a sampling rate of 256 Hz and referred to linked mastoids. A band-pass filter of 0.53 - 70 Hz and a notch filter of 50 Hz were used. Additionally, EOG and heart rate were assessed. Impedances were kept below 10 GΩ.

EEG data were processed using the Brain Vision Analyzer 2. Before analysis, the continuous EEG was (1) subjected to an Infinite response filter (IRR), (2) segmented in 2 s intervals, and (3) ocular movement correction was applied in accordance with Gratton et al. [19]. Furthermore, (4) an automatic artifact rejection was applied removing voltage steps greater than 50 µV/ms and amplitudes exceeding ±100 µV, followed by (5) a manual artifact rejection (e.g., checking for artifacts, focal abnormalities, drowsiness). A total of 25% of pre- and posttreatment EEGs, were co-rated by a co-author (RS). Visual inspection of the EEG segments showed major artifact to be caused by the pulse, therefore a pulse correction was used. A minimum of 30 artifact-free segments of the filtered EEG were required to be incorporated into the analysis.

Filtrated EEG data were Fourier-transformed with a Hanning window length of 20%, whereby theta (4 - 7 Hz), alpha (8 - 12 Hz), and beta (13 - 30 Hz) frequency bands were extracted. The extracted absolute power for each frequency band was aggregated to create an average for the central-frontal region (Cz, Fz, Fc1, Fc2), converted to relative band performance (%) related to the full spectrum of analyzed frequency bands (1 - 30 Hz), and ln-transformed to obtain normally distributed data before testing statistical hypotheses.

## 2. Supplementary Results

### Demographics and Pretreatment Values

The modified ITT sample (*N* = 38) had a mean age of 39.73 years (*SD* = 9.64, range: 21.25 - 56.33 years) and a mean BMI of 36.12 kg/m^2^ (*SD* = 5.06, range: 25.30 - 44.96 kg/m^2^). Participants experienced on average 4.32 OBEs over the last 14 days at baseline (*SD* = 3.19). While *n* = 36 participants had a full-threshold DSM-5 diagnosis of BED, *n* = 2 individuals had a DSM-5 diagnosis of BED of low frequency and/or limited duration, equally distributed among both neurofeedback groups. Further clinical and sociodemographic characteristics are presented in Table S2. In the food-specific paradigm, *n* = 10 individuals reported one or more mental comorbidities at baseline (depression *n* = 8, anxiety disorder *n* = 3, posttraumatic stress disorder *n* = 1, borderline personality disorder *n* = 1). Similarly, in the general paradigm *n* = 12 individuals reported one or more mental disorder in addition to BED at baseline (depression *n* = 8, anxiety disorder *n* = 2, posttraumatic stress disorder *n* = 1, borderline personality disorder *n* = 1, schizotypal personality disorder *n* = 1, personality disorder not further described *n* = 1).

### Feasibility and Acceptance

The study design specified a treatment duration of 42 days (6 weeks). The mean treatment duration of the food-specific and general paradigm was 58.0 days (*SD* = 14.40, *Mdn* = 47.00, range*:* 42 - 94 days) and 63.67 days (*SD* = 20.47, *Mdn* = 44.50, *range*: 43 - 117 days), respectively. Both EEG neurofeedback paradigms were well accepted, with the food-specific paradigm receiving a mean rating of 4.11, with higher scores indicating greater satisfaction (*SD* = 0.56, *Mdn* = 4.00, range: 1.90 - 5.00) and the general paradigm attaining a mean score of 3.74 (*SD* = 0.75, *Mdn* = 3.60, range: 2.80 - 5.00), without significant group differences, *F*(1, 31) = 2.16, *p* = .15, η^2^ = .07.

# Supplementary Tables

**Table S1**

*Consensus on the Reporting and Experimental Design of clinical and cognitive- behavioral Neurofeedback studies (CRED-nf) Checklist*

| Domain | Item # | Checklist item | Reported on page # |
| --- | --- | --- | --- |
| Pre-experiment | | | |
|  | 1a | Pre-register experimental protocol and planned analyses | 2, 3-5 |
|  | 1b | Justify sample size | 2 |
| Control groups | | | |
|  | 2a | Employ control group(s) or control condition(s) | 4-5 |
|  | 2b | When leveraging experimental designs where a double-blind is possible, use a double-blind | 2 |
|  | 2c | Blind those who rate the outcomes, and when possible, the statisticians involved | - |
|  | 2d | Examine to what extent participants and experimenters remain blinded | 2 |
|  | 2e | In clinical efficacy studies, employ a standard-of-care intervention group as a benchmark for improvement | - |
| Control measures | | | |
|  | 3a | Collect data on psychosocial factors | 3 |
|  | 3b | Report whether participants were provided with a strategy | 3 |
|  | 3c | Report the strategies participants used | - |
|  | 3d | Report methods used for online-data processing and artifact correction | 4 |
|  | 3e | Report condition and group effects for artifacts | - |

**Table S1 continued**

| Domain | Item # | Checklist item | | Reported on page # |
| --- | --- | --- | --- | --- |
| Feedback specifications | | | | |
|  | 4a | | Report how the online-feature extraction was defined | 3-5 |
|  | 4b | | Report and justify the reinforcement schedule | 3-5 |
|  | 4c | | Report the feedback modality and content | 3-5 |
|  | 4d | | Collect and report all brain activity variable(s) and/or contrasts used for feedback, as displayed to experimental participants | 3-5 |
|  | 4e | | Report the hardware and software used | 3-4 |
| Outcome measures | | | | |
| Brain | 5a | | Report neurofeedback regulation success based on the feedback signal | - |
|  | 5b | | Plot within-session and between-session regulation blocks of feedback variable(s), as well as pre-to-post resting baselines or contrasts | 7-9 |
|  | 5c | | Statistically compare the experimental condition/group to the control condition(s)/group(s) (not only each group to baseline measures) | 7-9 |

**Table S1 continued**

| Domain | Item # | | Checklist item | Reported on page # |
| --- | --- | --- | --- | --- |
| Behavior | 6a | | Include measures of clinical or behavioral significance, defined a priori, and describe whether they were reached | 7-9 |
|  | 6b | | Run correlational analyses between regulation success and behavioral outcomes | - |
| Data storage | | | | |
|  | 7a | Upload all materials, analysis scripts, code, and raw data used for analyses, as well as final values, to an open access data repository, when feasible | | - |

Note. Ros T, Enriquez-Geppert S, Zotev V, et al. Consensus on the reporting and experimental design of clinical and cognitive-behavioural neurofeedback studies (CRED-nf checklist). *Brain* 2020; 143: 1674–1685.

**Table S2**

*Sociodemographic Characteristics of the Modified Intent-to-Treat Sample*

| Baseline Characteristics | Food-specific neurofeedback  *n* = 19 | General  neurofeedback  *n* = 19 |
| --- | --- | --- |
|  | *M (SD)* | *M (SD)* |
| Age (years) | 41.39 (10.07) | 38.06 (9.16) |
| Body mass index (kg/m^2^) | 36.65 (5.71) | 35.60 (4.40) |
| Global eating disorder psychopathology | 3.06 (1.03) | 2.83 (1.08) |
| Depressive symptoms | 7.42 (3.27) | 9.11 (4.15) |
|  | *n* | *n* |
| Sex (female/male) | 15/4 | 13/6 |
| Education (low/high) ^a^ | 9/10 | 5/14 |

Note. *N* = 38., Depressive symptoms: short version of the Patient Health Questionnaire, Global eating disorder psychopathology: Eating Disorder Examination-Questionnaire.

^a^ low, ≤ 10 years of education, high, > 10 years of education.

**Table S3**

*Means and Standard Deviations for Outcome Variables of the Complete Case Sample*

| Variable | Food-specific neurofeedback (*n* = 13) | | | | General neurofeedback (*n* = 18) | | | | |  |
| --- | --- | --- | --- | --- | --- | --- | --- | --- | --- | --- |
|  | Baseline | Δ Pre-treatment | Δ Post-treatment | Δ 3 month | | Baseline | Δ Pre-treatment | Δ Post-treatment | Δ 3 month | |
| Clinical measures |  |  |  |  | |  |  |  |  | |
| Objective binge-eating episodes | 4.69 (4.15) | 0.15 (2.76) | -3.31 (4.48) | -2.62 (5.42) | | 4.56 (2.64) | -0.39 (3.82) | -2.17 (3.11) | -2.83 (4.60) | |
| Eating disorder psychopathology | 3.14 (1.13) | 0.06 (0.50) | -0.39 (0.86) | -0.39 (1.16) | | 2.89 (1.09) | -0.11 (0.58) | -0.16 (0.77) | -0.58 (0.85) | |
| Food craving | 59.77 (9.88) | 0.62 (11.12) | -11.00 (15.37) | -10.54 (23.89) | | 63.22 (11.49) | -4.61 (8.08) | -13.61 (10.27) | -19.83 (13.84) | |
| Self-efficacy ^a^ | 28.31 (5.34) | -0.46 (4.10) | -0.15 (2.97) | 0.58 (2.83) | | 24.44 (4.91) | 0.11 (3.14) | 1.39 (2.40) | 2.17 (3.20) | |
| Perceived stress | 48.59 (16.57) | 1.67 (18.67) | 0.64 (13.12) | -4.49 (12.66) | | 53.43 (19.14) | -1.39 (14.03) | -2.22 (18.96) | -2.88 (22.37) | |
| Depressive symptoms | 7.38 (3.62) | 0.77 (2.71) | -0.07 (3.07) | 0.23 (3.72) | | 9.28 (4.20) | 0.78 (4.98) | -0.67 (5.08) | 0.17 (6.22) | |
| Impact of weight on quality of life | 106.69 (16.12) | 5.08 (8.85) | 8.18 (11.89) | 5.94 (14.86) | | 106.76 (11.04) | -0.15 (7.68) | 3.25 (13.60) | 8.18 (13.24) | |
| Subjective impulsiveness | 30.39 (5.98) | 0.61 (2.68) | 0.68 (3.10) | 0.69 (3.17) | | 35.65 (5.80) | 1.52 (4.68) | 1.63 (6.09) | 1.24 (5.25) | |
| Body mass index | 37.19 (5.29) | 0.04 (0.56) | -0.35 (0.69) | -0.82 (1.12) | | 35.89 (4.32) | -0.99 (3.06) | -0.21 (1.18) | -0.60 (1.84) | |
| Waist-to-hip ratio | 0.86 (0.05) | 0.02 (0.02) | 0.02 (0.02) | 0.02 (0.02) | | 0.89 (0.09) | 0.00 (0.04) | 0.00 (0.06) | -0.01 (0.04) | |

**Table S3 continued**

| Variable | Food-specific neurofeedback (*n* = 13) | | | | | | | | | General neurofeedback (*n* = 18) | | | | | |  |
| --- | --- | --- | --- | --- | --- | --- | --- | --- | --- | --- | --- | --- | --- | --- | --- | --- |
|  | Baseline | Δ Pre-treatment | | | Δ Post-treatment | Δ 3 month | | | Baseline | | Δ Pre-treatment | Δ Post-treatment | | | Δ 3 month |  |
| Executive functions |  | |  |  | | |  |  | | |  | |  |  | | |
| Decision making ^a^ | -12.46 (31.65) | | -1.85 (31.21) | 4.67 (37.89) | | | 18.85 (38.79) | 8.33 (33.31) | | | 18 (42.2) | | 23.11 (30.76) | 24.33 (41.33) | | |
| Cognitive flexibility ^a^ | -2.97 (4.20) | | 0.55 (3.51) | 0.37 (3.89) | | | 2.44 (5.50) | -2.66 (8.92) | | | 2.25 (8.99) | | -1.26 (8.62) | -1.96 (9.12) | | |
| Impulsivity ^a^ | 0.48 (0.30) | | 0.07 (0.15) | 0.08 (0.11) | | | 0.10 (0.19) | 0.55 (0.25) | | | 0.09 (0.15) | | 0.09 (0.19) | 0.12 (0.16) | | |
| Planning ^a^ | 15.54 (3.55) | | 1.46 (3.80) | 0.54 (2.11) | | | 0.64 (3.60) | 17.39 (2.87) | | | 0.22 (2.82) | | 1.11 (3.08) | 0.10 (2.98) | | |
| Inhibitory control | 12.00 (8.29) | | -2.62 (7.38) | -3.69 (7.60) | | | -1.99 (9.21) | 10.44 (5.11) | | | -2.97 (3.31) | | -2.78 (5.04) | -2.57 (4.24) | | |
| Alertness | 2.06 (1.22) | | -0.08 (1.56) | -0.66 (1.20) | | | -0.37 (1.47) | 2.12 (1.33) | | | -0.45 (1.67) | | 0.01 (2.11) | -0.33 (1.65) | | |

Note. *N = 31.* OBEs: Δ Difference score of pre-, posttreatment, 3-month follow-up minus baseline. Objective binge-eating episodes: Eating Disorder Examination; eating disorder psychopathology: Eating Disorder Examination-Questionnaire (0 – 6* less favorable scores are asterisked); food craving: Food Cravings Questionnaire-trait-reduced (15 – 90*); self-efficacy: General Self-Efficacy Scale (10* - 49); perceived stress: Perceived Stress Questionnaire (0 – 100*); depressive symptoms: Patient Health Questionnaire-Depression (0- 27*); impact of weight on quality of life: Impact of Weight on Quality of Life-Lite (31 – 155*); subjective impulsiveness: Barratt Impulsiveness Scale (15 – 60*); decision making: Iowa Gambling Task; cognitive flexibility: Wisconsin Card Sorting Test; impulsivity: Delay Discounting Task, planning: Tower of London; inhibitory control: visual Go/NoGo paradigm.

^a^ positive difference values represent effects in expected direction.

**Table S4**

*Repeated Measures Univariate Analysis of Variance for the Complete Case Sample*

| Variable | Time | | | Group | | Time x Group | | | |
| --- | --- | --- | --- | --- | --- | --- | --- | --- | --- |
|  | *df* | *F* | η_p_^2^ | *df* | *F* | η_p_^2^ | *df* | *F* | η_p_^2^ |
| Clinical measures |  |  |  |  |  |  |  |  |  |
| Objective binge-eating episodes | 2, 36 | 9.01** | .24 | 1, 29 | 0.01 | .00 | 2, 36 | 0.79 | .03 |
| Eating disorder psychopathology | 2, 58 | 6.02** | .17 | 1, 29 | 0.03 | .00 | 2, 58 | 1.62 | .05 |
| Food craving | 2, 43 | 20.40** | .42 | 1, 29 | 1.62 | .05 | 2, 43 | 1.20 | .04 |
| Self-efficacy | 2, 51 | 2.53 | .08 | 1, 29 | 2.33 | .07 | 2, 51 | 0.35 | .01 |
| Perceived stress | 2, 46 | 1.23 | .04 | 1, 29 | 0.07 | .00 | 2, 46 | 0.54 | .02 |
| Depressive symptoms | 2, 58 | 2.40 | .08 | 1, 29 | 0.02 | .00 | 2, 58 | 0.20 | .01 |
| Impact of weight on quality of life | 2, 58 | 2.30 | .07 | 1, 29 | 0.56 | .02 | 2, 58 | 1.84 | .06 |
| Subjective impulsiveness | 2, 50 | 0.04 | .00 | 1, 29 | 0.30 | .01 | 2, 50 | 0.06 | .00 |
| Body mass index | 2, 43 | 0.11 | .00 | 1, 29 | 1.42 | .05 | 1, 43 | 0.91 | .03 |
| Waist-to-hip ratio | 2, 58 | 0.61 | .02 | 1, 29 | 3.73 | .11 | 2, 58 | 0.11 | .00 |

**Table S4 continued**

| Variable | Time | | | Group | | | Time x Group | | |
| --- | --- | --- | --- | --- | --- | --- | --- | --- | --- |
|  | *df* | *F* | η_p_^2^ | *df* | *F* | η_p_^2^ | *df* | *F* | η_p_^2^ |
| Executive functions |  |  |  |  |  |  |  |  |  |
| Decision making | 2, 58 | 2.47 | .08 | 1, 29 | 1.56 | .05 | 2, 58 | 0.84 | .03 |
| Cognitive flexibility | 2, 51 | 10.98** | .28 | 1, 29 | 0.55 | .02 | 2, 51 | 5.99** | .17 |
| Impulsivity | 2, 45 | 0.56 | .02 | 1, 29 | 0.01 | .00 | 2, 45 | 0.03 | .00 |
| Planning | 2, 58 | 0.59 | .02 | 1, 29 | 0.17 | .01 | 2, 58 | 1.70 | .06 |
| Inhibitory control | 2, 58 | 0.70 | .02 | 1, 29 | 0.00 | .00 | 2, 58 | 0.49 | .02 |
| Alertness | 2, 58 | 0.07 | .00 | 1, 29 | 0.05 | .00 | 2, 58 | 2.30 | .07 |

Note. *N =* 31*.* Objective binge-eating episodes: Eating Disorder Examination; eating disorder psychopathology: Eating Disorder Examination-Questionnaire; food craving: Food Cravings Questionnaire-trait-reduced; self-efficacy: General Self-Efficacy Scale; perceived stress: Perceived Stress Questionnaire; depressive symptoms: Patient Health Questionnaire-Depression; impact of weight on quality of life: Impact of Weight on Quality of Life-Lite; subjective impulsiveness: Barratt Impulsiveness Scale; decision making: Iowa Gambling Task; cognitive flexibility: Wisconsin Card Sorting Test; impulsivity: Delay Discounting Task, planning: Tower of London; inhibitory control: visual Go/NoGo paradigm.

* *p* < .05; ** *p* < .01

# References

[1] Hilbert A, Tuschen-Caffier B. Eating Disorder Examination- Questionnaire 2 Deutschsprachige Übersetzung. 2. Auflage. Tübingen: DGVT Verlag, 2016.

[2] Hilbert A, Tuschen-Caffier B. Eating Disorder Examination: Deutschsprachige Übersetzung [Eating Disorder Examination: German translation]. 2. Auflage. Tübingen: DGVT Verlag, 2016.

[3] Meule A, Lutz A, Vögele C, et al. Food cravings discriminate differentially between successful and unsuccessful dieters and non-dieters. Validation of the Food Cravings Questionnaires in German. Appetite 2012; 58: 88–97.

[4] Schwarzer R, Jerusalem M. Generalized Self-Efficacy scale. In: Weinman J, Wright S, Johnston M (eds) Measures in health psychology: A user’s portfolio. Causal and control beliefs. Windsor, England: NFER-NELSON, 1995, pp. 35–37.

[5] Fliege H, Rose M, Arck P, et al. Validierung des “Perceived Stress Questionnaire“ (PSQ) an einer deutschen Stichprobe. Diagnostica 2001; 47: 142–152.

[6] Gräfe K, Zipfel S, Herzog W, et al. Screening psychischer Störungen mit dem “Gesundheitsfragebogen für Patienten (PHQ-D)“. Diagnostica 2004; 50: 171–181.

[7] Mueller A, Holzapfel C, Hauner H, et al. Psychometric Evaluation of the German Version of the Impact of Weight on Quality of Life-Lite (IWQOL-Lite) Questionnaire. Exp Clin Endocrinol Diabetes 2011; 119: 69–74.

[8] Meule A, Vögele C, Kübler A. Psychometric evaluation of the German Barratt Impulsiveness Scale – Short Version (BIS-15). Diagnostica 2011; 57: 126–133.

[9] Bechara A, Damasio AR, Damasio H, et al. Insensitivity to future consequences following damage to human prefrontal cortex. Cognition 1994; 50: 7–15.

[10] Heaton RK, Chelune GJ, Talley JL, et al. Wisconsin Card Sorting Test manual. Odessa, Florida: Psychological Assessment Resources, Inc, 1993.

[11] Richards JB, Zhang L, Mitchell SH, et al. Delay or probability discounting in a model of impulsive behavior: effect of alcohol. J Exp Anal Behav 1999; 71: 121–143.

[12] Myerson J, Green L, Warusawitharana M. Area under the curve as a measure of discounting. J Exp Anal Behav 2001; 76: 235–243.

[13] Kaller CP, Unterrainer JM, Kaiser S, et al. Wiener Testsystem: Tower of London-Freiburger Version. Mödling: Schuhfried, 2015.

[14] Schuhfried. Vienna test system. Moedling: Schuhfried Gmbh, 2015.

[15] Kaiser S, Aschenbrenner S, Pfüller U, et al. Wiener Testsystem: Response Inhibition. Mödling: Schuhfried, 2015.

[16] Lezak MD, Howieson DB, Bigler ED, et al. Neuropsychological Assessment. New to this Edition: Oxford, New York: Oxford University Press, 2012.

[17] Sturm W. Wiener Testsystem: Wahrnehmungs- und Aufmerksamkeitsfunktionen. Mödling: Schuhfried, 2015.

[18] Blechert J, Meule A, Busch NA, et al. Food-pics: an image database for experimental research on eating and appetite. Front Psychol 2014; 5: 617.

[19] Gratton G, Coles MGH, Donchin E. A new method for off-line removal of ocular artifact. Electroencephalogr Clin Neurophysiol 1983; 55: 468–484.
